# Assessment of depression and anxiety in young and old with a question-based computational language approach

**DOI:** 10.1038/s44184-023-00032-z

**Published:** 2023-07-24

**Authors:** Sverker Sikström, Bleona Kelmendi, Ninni Persson

**Affiliations:** 1 Department of Psychology, Lund University, Lund, Sweden; 2 Department of Psychology, Potsdam University, Potsdam, Germany; 3 Department of Psychology, Uppsala University, Uppsala, Sweden; 4 Institute for Globally Distributed Open Research and Education (IGDORE), Gothenburg, Sweden

**Keywords:** Human behaviour, Health care, Psychology

## Abstract

Middle aged adults experience depression and anxiety differently than younger adults. Age may affect life circumstances, depending on accessibility of social connections, jobs, physical health, etc, as these factors influence the prevalence and symptomatology. Depression and anxiety are typically measured using rating scales; however, recent research suggests that such symptoms can be assessed by open-ended questions that are analysed by question-based computational language assessments (QCLA). Here, we study middle aged and younger adults’ responses about their mental health using open-ended questions and rating scales about their mental health. We then analyse their responses with computational methods based on natural language processing (NLP). The results demonstrate that: (1) middle aged adults describe their mental health differently compared to younger adults; (2) where, for example, middle aged adults emphasise depression and loneliness whereas young adults list anxiety and financial concerns; (3) different semantic models are warranted for younger and middle aged adults; (4) compared to young participants, the middle aged participants described their mental health more accurately with words; (5) middle-aged adults have better mental health than younger adults as measured by semantic measures. In conclusion, NLP combined with machine learning methods may provide new opportunities to identify, model, and describe mental health in middle aged and younger adults and could possibly be applied to the older adults in future research. These semantic measures may provide ecological validity and aid the assessment of mental health.

## Introduction

Depression and anxiety disorders are global phenomena and create widespread and growing problems in healthcare^1^. Untreated depression can be disabling^2–5^ and have financial consequences^6^. In 2000, the economic burden of depression in the US was an estimated USD 83.1 billion, of which USD 51.5 billion were workplace costs^7^. Early and efficient diagnostic methods are essential for applying effective and appropriate treatment. The development of more precise diagnostic instruments and accessible treatment methods is warranted. One important aspect is how such disorders vary across the lifespan. Rating scales have typically been used to quantify levels of depression and anxiety. In contrast, language is a natural way for people to communicate their mental states, and language ability is preserved or even improves as people age^8^. Recent advancements in computational language models (CLA) allow for quantitative assessment of depression and anxiety using words generated from open questions related to mental health^9^. This unique study aims to assess age differences in the reporting of mental health issues using question based computational language assessments (QCLA), which to the best of our knowledge has not been done previously.The prevalence of depression and anxiety varies across the lifespan^10,11^, therefore the age dependent differences in the word responses and description of mental health using the QCLA approach is of interest. Studies have identified age differences in the prevalence of depression and anxiety. Younger adults (16–29 years) were more likely to be affected by depression and severe anxiety than the older adults^10^. Contrary to this report, Lenze et al.^12^, found a relatively high rate of both current and lifetime anxiety disorders in the elderly, where 35% of the older participants had received an anxiety disorder diagnosis at least once, and 23% had been diagnosed recently. In summary, the prevalence of depression and anxiety disorders varies across the lifespan.

In the following, we will provide a current review of the literature on the differences in terms of mental health between younger, middle age and old adults.

### Young adults

Younger adults (16–29 years) are more likely to be affected by depression and severe anxiety than older adults^10^. In 2022, the young age group was most affected by severe anxiety at 16% and in Sweden^13^; 4% was diagnosed with depression^13^. There is emerging evidence, that the prevalence of anxiety disorders is associated with young age, but also female gender and given chronic diseases^14^. In terms of aetiology, different subtypes of childhood maltreatment, child–parent bonding, stressful life events, as well as a genetic liability predict subsequent depression^15,16^. Depression is a risk factor for all-cause mortality, with greater risk for greater severity^17^. Thus, suicide is the most common cause of death in young men in the United Kingdom aged between 25 and 34 years^18^. Life changes and stress because of the Covid-19 pandemic are mirrored in an increase of depression and anxiety in the young^19^. Younger adults who struggle financially are at higher risk of mental health problems^20^.

### Middle-aged adults

In Sweden, approximately 7% of middle-aged adults (30–59 years) are diagnosed with depression^13^, while only very few are affected by severe anxiety^13^. Regarding the period prevalence of 1 year, one in seven middle-aged participants (45–64 years) experienced symptoms consistent with ICD-10 anxiety or affective disorder in the preceding 12 months^21^. Anxiety disorders are most prevalent in the lifespan of 25–44 years^13^. In comparison to the prevalence of 1 in 16 of the older age group (60–75 and older), middle-aged adults were more likely to be affected by anxiety and affective disorders^21^. Major depressive disorder (MDD) is a common mental illness that may occur at any age during the lifespan. However, the highest risk period for onset is from mid to late adolescence to early 40s^22^. The presence of a physical disorder is significantly associated with the presence of mental disorders for middle-aged people^21^. Depression may even worsen health conditions, as it is associated with macrovascular complications and all-cause mortality in a patient cohort with diabetes^23^. For anxiety disorders among middle-aged and older adults, physical health, socioeconomic status, immigrant status and nutritional factors are associated with its occurrence^24^. Perceived stress interacts with age during the development of depression and anxiety disorders^25,26^. Employment and marital status may function as an important predictor of mental disorders in middle-aged groups^21^. Middle-aged participants were more likely to be affected by a mental disorder 12 months after experiencing separation, divorce, or death of a partner^13,21^.

### Old adults

Regarding the point and lifetime prevalence of anxiety disorders in the elderly, Lenze et al.^12^ found a relatively high rate of both current and lifetime anxiety disorders in the elderly, where 35% of the older participants had received an anxiety disorder diagnosis at least once, and 24% had been diagnosed recently. Depression late in life displays a clinical phenomenon^27^; there is a greater likelihood of comorbidities, differing aetiology and symptom expression compared to depression in younger adults. The aetiology of depression in the elderly is more heterogeneous than in younger adults^28^. Age-related changes in the brain, neurodegenerative and cardiovascular diseases may be of importance for the development of depression in later life^27,28^. Studies have shown that comorbidity between clinically significant depression and anxiety may be as high as 48.3%^29^. The risk of mortality due to depression and anxiety disorders is higher in older adults^30^, while suicide risk is particularly high in older men^31^. For the elderly (75 years or older), the likelihood for a suicide attempt rises by three times in comparison to younger age groups^32^. Anxiety-related disorders are also correlated with a higher level of suicidality^12^. The elderly showed higher levels of loneliness, as well as higher levels of distress and exhaustion during the Covid-19 pandemic, with anxiety influencing the emergence of depression^33^. Bergdahl and Bergdahl^25^ observed perceived stress to be impacting the development of depression and anxiety disorders among high age groups (60–69 years) in Sweden. Elderly are more likely to be widowed and in poor health compared to younger adults, which can aggravate the risk of depression^34,35^. In contrast, social capital (i.e., resources from social networks) may function as a source of mental wellbeing in the elderly^36^.

In summary, the prevalence of depression and anxiety disorders varies across the lifespan. While there are no age of onset (AOO) specific guidelines for treating depression, the treatment of pre-adult or late-life depression should be considered individually depending on the patient^22^, as age-specific differences in life circumstances may influence the onset. Therefore specialised diagnostic methods should be considered for younger and older adults implementing each reality of life and language for patients affected by depression and anxiety disorders.

Artificial intelligence (AI) technologies have shown beneficial effects in clinical decision-making, treatments, managing healthcare and research^37–39^. AI technologies can help quantify mental health in electronic health records, mood rating scales, brain imaging data, novel monitoring systems, smartphone or video data and social media platforms. AI has demonstrated great accuracy in predicting and classifying depression, anxiety and other psychiatric illnesses or suicide ideation^40,41^. AI methods have been used to analyse social media posts for depression, providing an opportunity for studying a large population^42,43^ using probabilistic models, crowdsourcing technology^44,45^ and computational language assessments (CLA) (Eichstaedt et al.^6^). These findings suggest the significance and value of words when describing mental health.

A natural language processing method called latent semantic analysis (LSA)^46^, where open-ended questions about mental health are applied, may facilitate registration of information closer to individual behaviour in a real-world setting. The LSA has been validated against several traditional rating scales, and demonstrated good statistical properties with competitive, or even higher reliability^9^.

The QCLA can be applied to semantic data (i.e., words and sentences), where the assessment is based on high-dimensional word embeddings from a large language corpus^47^). Kjell et al.^48^ investigated word response relating to the symptoms of major depressive disorder (MDD) and generalised anxiety disorders (GAD) as described in the Diagnostic and Statistical Manual of Mental Disorders (DSM-5). The results of the QCLA showed that all primary and secondary language responses correlated significantly with the depression scale Patient Health Questionnaire 9-item (PHQ-9^49^). Together, these findings suggest that QCLA may be helpful in clinical assessment of mental health.

Machine learning (ML) and (AI) methods demonstrate potential, as subjective descriptions of mental health can be monitored and to facilitate the diagnostic process^50–53^. Advances in ML and AI could provide more personalised care for patients to aid decisions on the best suitable treatments and interventions^54^. While text offers a rich source of unstructured information for ML models, there is risk that this learning will also pick up the human biases that ML is based on ref. ^54^. An example of such bias is that old and young people may be assessed on the same criteria, whereas symptoms may differ with age, which emphasises that more research is required.

Currently, there is a large gap in knowledge about how people of different ages describe their mental health in their own words. An age-specific application of machine learning and artificial intelligence methods may allow for personalised assessment and treatment of mental health^55,56^.

Our *research question* addresses differences in descriptive word responses related to mental health in younger and middle-aged adults. The aim is to investigate potential differences in the semantic representation across the lifespan.

We hypothesise that the semantic representations of mental health differ for younger (i.e., young) and older (i.e., middle-aged) adults (H1), and that these differences are expressed in specific semantic attributes (H2). Given H1 is supported, we hypothesise that different prediction models are required for predicting mental health in younger and older adults (H3). Due to language skills improving with age, we hypothesise that the prediction models may be more accurate for older (H4). Given previous reports on rating scales for measuring mental health in younger and older adults, we hypothesise that the language-based prediction models of older individuals show better mental health than for the younger (H5).

## Methods

### Participants

The study consisted of 883 participants with English as a first language. Seven participants were removed from the analysis as they either failed to follow the instructions, or did not respond to the control questions correctly (e.g., choose the option on the left hand side). The final analysis included 876 participants. 457 participants were recruited from the Mechanical Turk (www.mturk.com) platform, and 419 from the Prolific Academic (https://prolific.co/) platform. Half the participants were recruited by screening for MDD or GAD as assessed by using the self-reported depression and anxiety symptoms (SDAS) (Sikström et al.,^57^ in revision), which is an online version of the Mini International Neuropsychiatric Interview (MINI). The SDAS has been validated by clinicians for MDD (Kappa = 0.76) and for GAD (Kappa = 0.52), for details of this see the Supplementary Information. The other half of participants were recruited without screening; however, they were also assessed by SDAS. Using this measure, 61 (34 younger) participants had MDD alone, 137 (70 younger) had GAD alone, and 259 (139 younger) had both MDD and GAD. Participants younger than the median age of 32.5 were categorised as younger. The age in the given sample ranged from 18 to 70 years (*M* = 35.5, SD = 11.9). 538 participants identified as female, 327 as male and 11 as “other gender”. The study lasted approximately 20 min, and participants received USD 4 for their time.

### Material

#### Semantic open-ended questions—Word responses

In total, the participants were asked 11 open-end questions and five rating scales. The open-ended questions can roughly be categorised into topics of; mental health, causes of mental health, positive psychology, and symptoms of mental health. Three open-ended questions were about mental health: “Describe your mental health with descriptive words”, “During the last two weeks, describe in words whether you felt depressed or not”, “During the last two weeks, describe in words whether you have felt worry or not”. They were also asked three questions about the underlying causes of their mental health, depression, and anxiety (“Describe the reason for your mental health/depression/worry in descriptive words). There were two open-ended questions for positive psychology, one on satisfaction (“Overall in your life, describe in words whether you are satisfied or not?”) and harmony (“Overall in your life, describe in words whether you are in harmony with your life or not?”). Eight questions were asked about symptoms (“Describe your sleep/concentration/appetite/energy/self/movement/behaviour/interest with descriptive words)”. The participants were asked to respond using five words for the mental health questions (general, depression, anxiety), three words for the reason questions (general reason, depression reason, anxiety reason), three words for the positive psychology questions (satisfaction, harmony), and two words for the symptom questions. The participants were asked to write one word in each text box, thus the number of boxes matched the number words they were asked to write.

##### Rating scales

The following rating scales were used to measure depression PHQ-9^49^, anxiety Generalised Anxiety Disorder 7-item scale (GAD-7^58^)), satisfaction with life (SWILS^59,60^), and harmony in life (HILS^48^). SDAS was used to validate the participants’ MDD and GAD diagnoses.

##### Control items

One control item per rating scale was included, for example “Answer ‘disagree’ on this question”. If the participant failed to answer all the control questions correctly, they were excluded from the analysis. These control questions were essential for ensuring the quality of the dataset by guaranteeing the participant’s focus on the task and to improve the statistical reliability^61–63^.

##### Demographic inventory

A demographic survey was included, in which the participants were asked about their age and gender. They were also asked to provide their country of origin and first language, as well as a description of their estimated household income. In order to measure the estimated household income, the participants responded to the question “Does the total income of your household allow you to cover your needs?” with either, “Our income does not cover our needs, there are great difficulties” (1) to “Our income covers our needs, we can save” (7).

### Procedure

To participate in the study, a declaration of informed written consent was required. Participants were told that their responses would be anonymised before analysis, and that they could withdraw from the study at any time without needing to give a reason. The questions and rating scales were presented in a random order. Finally, demographic information was collected, and a debrief on the purpose of the study was provided.

### Ethics

The study was reviewed by the Swedish Ethical Review Authority (EPN), who determined no ethical approval was needed, as the participants were anonymously recruited and tested (reg. no.: 2020-00730).

### Data analyses

The primary aim of the analysis was to study age differences in mental health by looking at the differences in the semantic representations of the descriptive words dependent on their age. The machine learning was trained to the continuous value of age. Methods proposed by Kjell et al.^9^ were used and the words were quantified using a latent semantic analysis (LSA) trained to predict the participants’ age with machine learning.

The data analysis was conducted using the online software for statistical analysis of text, SemanticExcel.com. This software includes pre-programmed semantic representations that are generated by the LSA method based on the English version of Google N-gram data (*N* = 5). In this method, a co-occurrence matrix is generated first, where each cell includes the frequency of a word in the N-gram. The content of the cells is then normalised by taking the logarithm of the frequency plus one. A semantic representation is then generated by applying a data compression algorithm known as the singular value decomposition (SVD). This generates vectors describing the words in the corpus. Each vector consists of 512 dimensions and is normalised to a length of one. The word responses were added together, and the length was again normalised to one, so that each response to a word question was described by one vector (see Kjell et al.^9^ for details). The semantic similarity between two semantic representations can be measured using the cosine of the angle between the vectors, which is calculated as the inner product of the two vectors divided by the product of their magnitudes.

We investigated whether semantic representation depends on age by predicted age from the semantic representation. A variable, called “All texts”, were generated that included the text responses from all the questions for each participant. Age was predicted based on this variable, using the method described in the “Data analysis” section.

Given that the semantic representation differs depending on age, we are interested in studying what attributes are indicative of younger and older people’s description of their mental health (where participants younger than the median age of 32.5 were categorised as young). We used the model generated for the concatenation of all the text that was generated in the analysis of H1, and applied this model to words in the dataset. Then we used two-sided *t* tests to investigate whether each word was indicative of young or old participants.

We applied the linguist inquire word count (LIWC), a method to assess the how related texts are to certain predefined and manually generated word list^64^. These word lists (*N* = 63) represent psychologically relevant categories of words (e.g., emotions, work, stress). The LIWC measures is based on word frequency, and not on word embeddings, and is calculated by counting the percentage of words in each text that is also is presented in each LIWC word list.

Machine learning was used to study whether the semantic representation depended on the age of the participants (for methodological details, see Kjell et al.^9^). Multiple linear regression (*y* = *c* × *x*) was used to predict the age (*y*) using the semantic representation (*x*) as input. The training and test data set was separated by using a 10% leave-out cross-validation procedure. The number of dimensions used in the regression was optimised using a nested cross-validation procedure. The predicted values of age were compared with the empirical data using Pearson correlation (*r*), and the proportion of explained variance (*r*^2^).

## Results

### Basic statistics

The dataset consisted of a total of 36,396 words, with 4010 unique words. Participants on average generated 42 words (standard deviation 1.4). The mean natural word frequency, as measured by Google N-grams, was 0.00011. The frequency of the words, nor the log frequency of the words, did not correlate with age.

### H1: does the semantic representation depend on age?

The results showed that this semantic representation from the All texts variable predicted age (Pearson correlation between predict and empirical age; *r* = 0.31, *r*^2^ = 0.10, *p* < 0.0001). Furthermore, prediction models were generated separately for each text variable. The results showed that seven variables were significant, following Bonferroni correction for multiple comparison (sleep, self, affective behaviour, general, energy, harmony, depression), gender without correction for multiple comparison (movement, worry, depression reason, worry reason). Three questions did not correlate with age (appetite general, and interest) (see Table 1).

**Table 1:** Pearson correlations between the semantic questions trained to the variable age.

Label	*r*	*p*	*r*^2^	RMSE	Min	Max
All texts	0.310**	<0.0001	0.096	12.03	35.65	62.25
Sleep	0.191**	<0.0001	0.036	14.08	35.46	103.19
Self	0.170**	<0.0001	0.029	11.73	35.50	46.58
Affect behaviour	0.151**	<0.0001	0.023	15.48	35.79	83.53
General reason	0.137**	<0.0001	0.019	11.93	35.46	49.97
Energy	0.122**	<0.0001	0.015	11.93	35.48	49.18
Harmony	0.116**	0.0003	0.013	11.98	35.51	47.86
Depression	0.098**	0.0019	0.010	12.44	35.42	54.63
Movement	0.087*	0.0054	0.008	12.81	35.43	70.70
Worry	0.083*	0.0072	0.007	15.72	34.77	89.05
Depression, reason	0.072*	0.0161	0.005	12.36	35.46	72.90
Worry, reason	0.071*	0.0181	0.005	13.24	35.33	58.09
Satisfaction	0.069*	0.0202	0.005	12.56	35.17	70.00
Concentration	0.065*	0.0272	0.004	12.15	35.51	53.12
Appetite	0.005	0.4403	0.000	13.04	35.63	60.71
General	−0.007	0.5822	0.000	12.48	35.59	51.91
Interest	−0.011	0.6313	0.000	13.78	35.93	92.32

*p  <  0.05 uncorrected for multiple comparisions, **p < 0.05 following Bonferoni correction for multiple comparisions.

Note. The rows show the label of the open-ended questions, the Pearson correlation coefficients (*r*), the *p* values, root mean squared error (RMSE), minimum predicted age (Min), and maximum predicted age (Max). The *p* value states the probability of observing a correlation at least as large (in absolute terms) as the observed correlation under the assumption that the true correlation is 0.

### H2: word indicative of younger and older adults

**Fig. 1:**
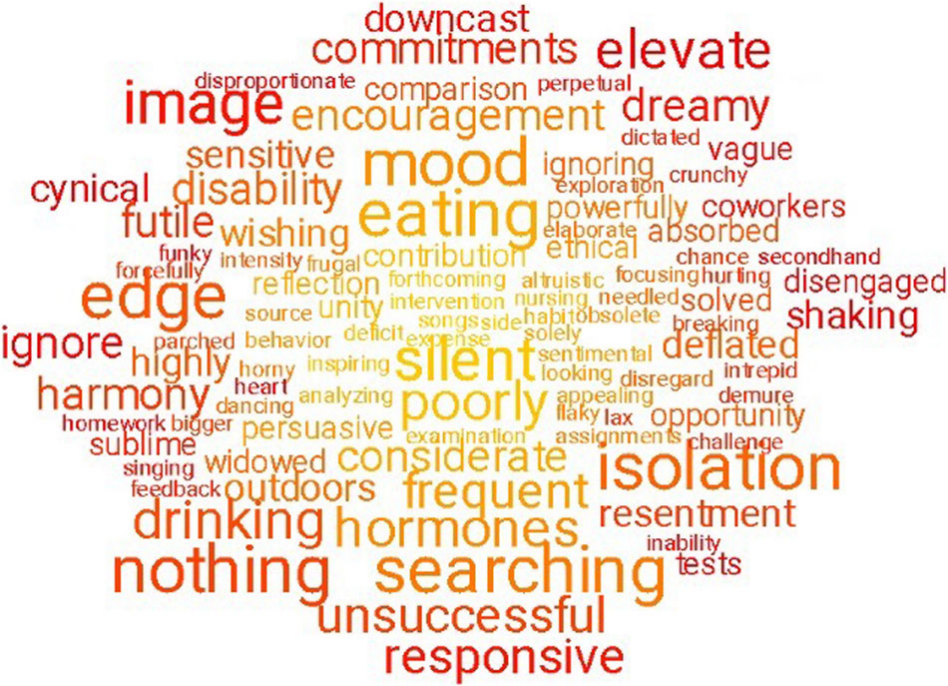
Word clouds summarising the text data. Note: The word clouds show 100 words that are the most indicative of the text data compared with a random sample of words in Google N-gram. The words were taken from the concatenation of all text questions “Text all” and compared with a random sample of words in Google N-gram, using the multiple linear regression as specified in the text. All words showed significant Pearson correlations with age following the Bonferroni correction for multiple comparisons, where the colour coding represent the *p* values. The font size represents the frequency of the words in the data set.

**Fig. 2:**
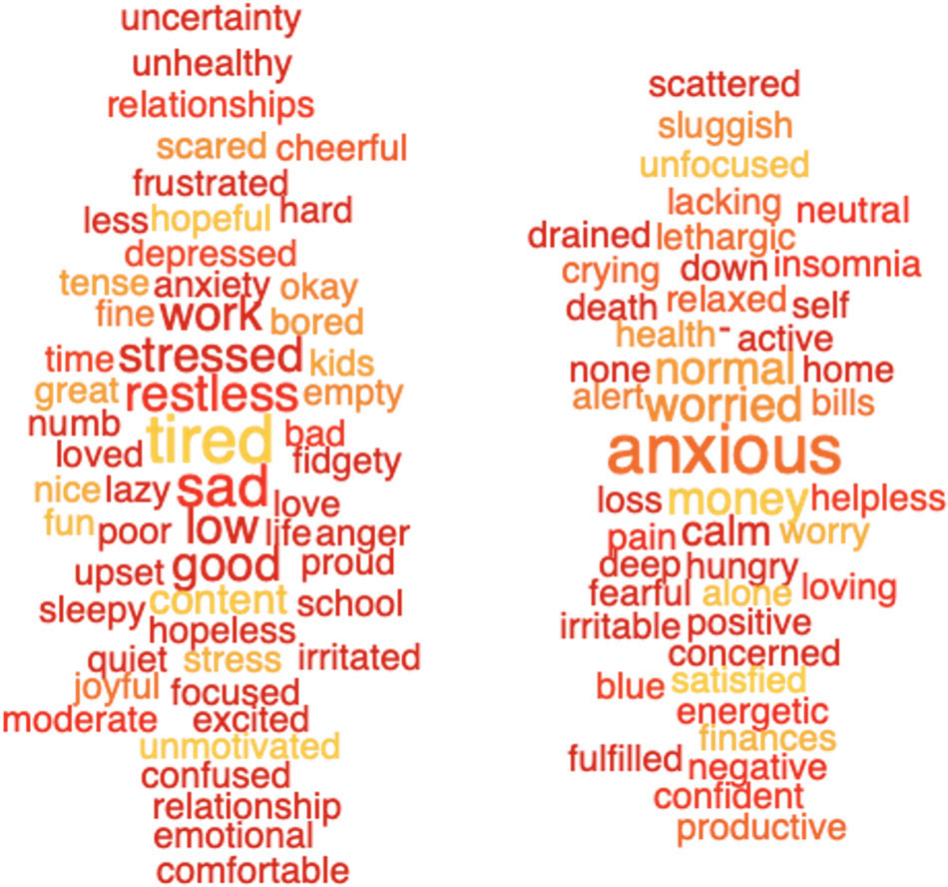
Word clouds indicative of young (left) and old (right). Note: The word clouds show 100 words that are the most indicative of the younger adults (left cloud) and older adults (right cloud) ages. See also footnote to Fig. 1.

Figure 1 shows a word cloud that summarise the words for all participants (see the footnote for details). Figure 2 shows word clouds indicative of young (left) and old (right) participants and follows the Bonferroni correction for multiple comparisons. These words were manually classified by the authors into ten semantic categories in Table 2A.

**Table 2:** (A) Words indicative of age manually classified into ten semantic categories; (B) LIWC categories indicative of younger and older age.

A			
Category	Young	Old	
BODY/ENERGY		Crying, insomnia, death, pain, hungry, health, lethargic, sluggish	
WORK	School, work		
RELATIONSHIP	Kids, relationship, relationships	Alone	
MONEY	Poor	Finances, bills, money	
DEPRESSION	Depressed, hopeless, frustrated, bored, empty, sad, tired, low, unmotivated, less, sleepy	Blue, helpless, negative, down, loss	
ANXIETY	Scared, tense, anxiety, irritated, anger, uncertainly, emotional, unhealthy, confused	Worried, anxious, worry, fearful, concerned, irritable, scattered	
STRESS	Stress, stressed, restless, upset, time, fidgety		
POSITIVE EMOTIONS	Hopeful, fun, comfortable, joyful, excited, okay, fine, good, proud, great, content, loved, nice, cheerful	Calm, confident, loving, productive, alert, energetic, positive, fulfilled, satisfied, relaxed, active	
OTHER	Hard, numb, bad, lazy, quiet, focused	Unfocused, lacking, drained, deep	
NEUTRAL	Moderate, life	Normal, none, neutral, self	
B			
**LIWC**	**Old/young**	***p***	***r***
Insight	Old	0.0001**	0.1312
Cognitive processes	Old	0.0002**	0.1263
Family	Old	0.0062*	0.0923
Money	Old	0.0110*	0.0858
Discrepancy	Old	0.0224*	0.0771
Positive emotion	Old	0.0398*	0.0695
Anxiety	Young	0.0429*	−0.0684
Friends	Young	0.0276*	−0.0745
Function words	Young	0.0123*	−0.0846
Adverbs	Young	0.0115*	−0.0853
Space	Young	0.0061*	−0.0926
Assent	Young	0.0026*	−0.1017
Negative emotion	Young	0.0022*	−0.1033
Feeling	Young	0.0018*	−0.1052
Relativity	Young	0.0018*	−0.1055

Note: The columns show the LIWC categories with significant Pearson correlation with age (*p < 0.05; **p < 0.05 following the Bonferroni correction for multiple comparison), *p* values that the correlations differ from zero, and the correlation coefficient (*r*).

The results show that older people relate their mental health to words related to anxiety (“anxious”, “worry”, etc.), whereas young individuals focus on words related to depression and stress (“sad”, “stressed”, “restless”, “depressed” etc.). Furthermore, younger adults mention issues related to their main activities (e.g., “work”, “school”, “relationships”), whereas the older population uses words more focused on feelings and body states (i.e., “hunger”, “health”, “death”, “crying”, “insomnia”).

Here we used LIWC to investigate which categories are indicative of the younger and the older groups by using the “All text” variable. The LIWC scores in the 63 categories was correlated with age. Table 2B shows the LIWC categories with Pearson correlation coefficients that were significantly different from zero. The “insight” and “cognitive processes” categories correlated positively with age, following the Bonferroni correction for multiple comparisons. The “family”, “money”, “discrepancy” and “positive emotion” categories also correlated positively, but without correction for multiple comparisons. The “anxiety”, “friends”, “function words”, “adverbs”, “space”, “assent”, “negative emotion”, “feeling” and “relativity” categories correlated negatively with age, without correction for multiple comparisons.

### H3: do younger and older adults require different semantic prediction models?

Here we investigate whether a prediction model of mental health trained on older or younger adults differs from a prediction model applied to younger or and older groups. Two hypotheses are tested here. If the prediction models that are trained and tested on the older group are better at predicting mental health scores than the prediction models that are trained and tested on the younger group, then this supports the idea that the data quality of the old group is better than the younger group (H3). Furthermore, if there is an interaction effect between whether the training and test is made on the same versus different groups, and the older versus younger group, then this supports the hypothesis that different prediction models are required for older versus the younger groups (H2).

Hypothesis 3 is evaluated as follows: the data set was divided using median split criteria, where young participants were aged below 32.5, and an older group equal to or larger than this age. The cross-validation procedure was applied separately to each of the 17 semantic representations (listed in Table 1). These semantic representations were trained on each of the four mental health rating scales (i.e., related to depression (PHQ-9), anxiety (GAD-7), harmony (HILS) and satisfaction (SWLS)). 68 prediction models were generated for each of the four groups and the results were evaluated using Pearson correlations between predicted and empirical rating for each of these models.

**Fig. 3:**
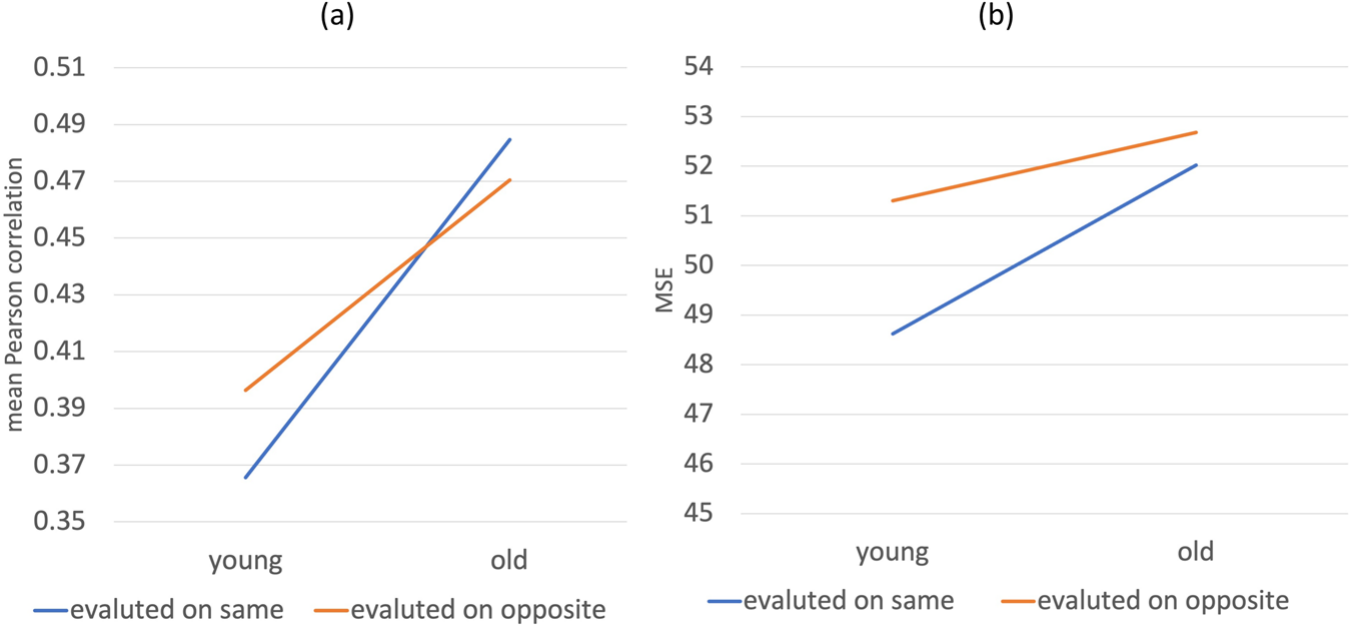
Mean Pearson correlation and MSE depending on training or testing on older or younger participants. Note: The y-axis shows the Pearson correlation (**a**) and mean squared error (MSE) (**b**) between predicted and empirical rating scales averaged over all the semantic representations and the rating scales. The training data is divided into younger (left) and older participants (right), using either models trained on the same data set that they were applied on (blue) or trained on the opposite dataset (red).

A repeated measure ANOVA was used to analyse the correlation coefficients, where the factors were age (younger versus older) and testing-training (same versus opposite data). There was a significant age by test–training interaction (*F*(1, 67) = 22.2, *p* < 0.001, Fig. 3), indicating that models generated older and younger people depending on whether they were tested on the younger and older participants. This suggests that the different prediction models are required for the younger and older study groups, and Hypothesis 3 is supported.

### H4: do older people generate better semantic prediction models?

The ANOVA also shows a significant main effect on age (*F*(1, 67) = 196.3, *p* < 0.001) indicating that ratings scales are better predicted from the semantic representations for the older compared to the younger participants (Fig. 3), supporting Hypothesis 4. Thus, accuracy was higher for older participants both when they were evaluated on the older participants and when they were evaluated on the younger participants.

### H5: mental health in younger and older adults

**Fig. 4:**
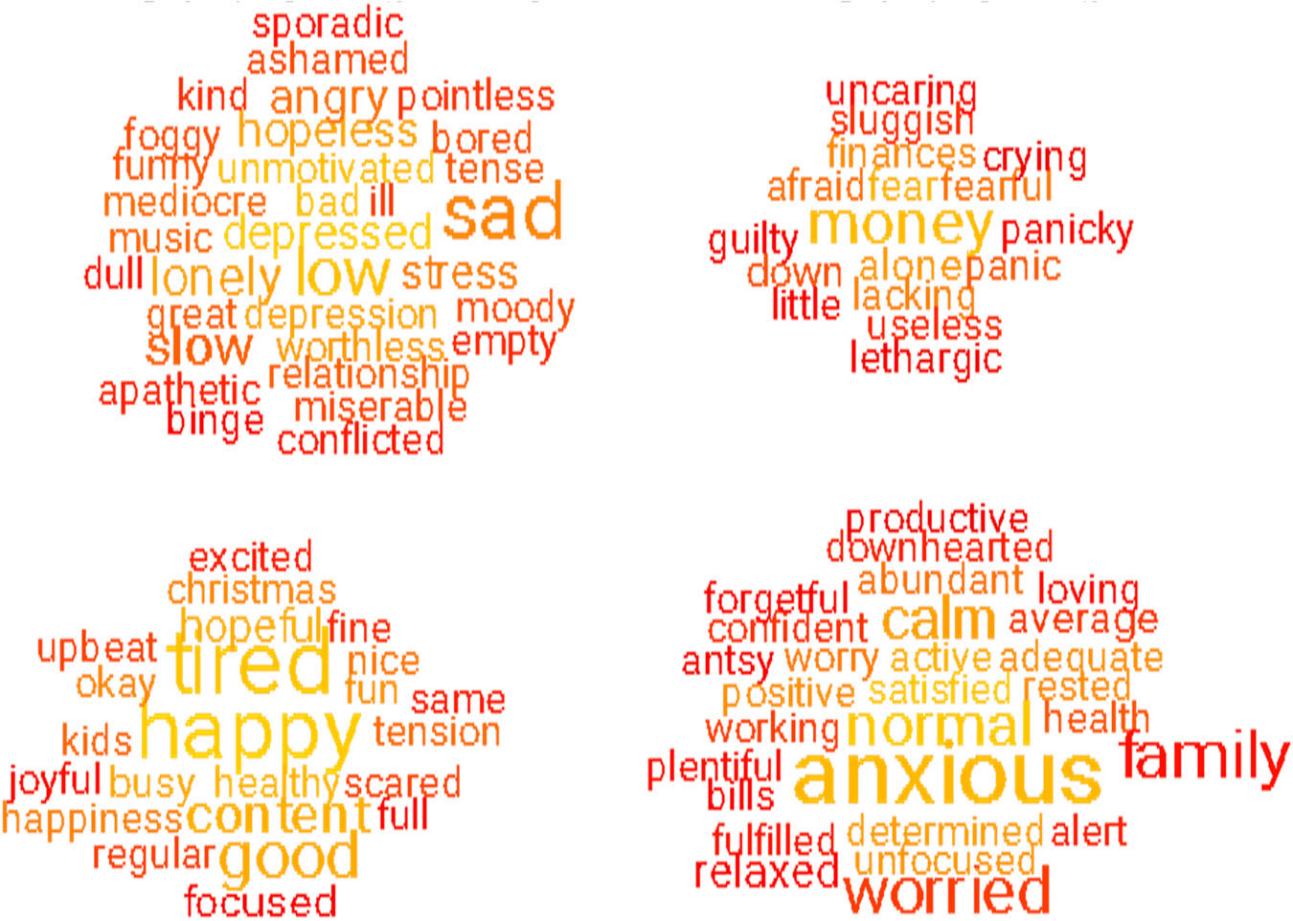
Word clouds indicative of young and old participants (*x*-axis) and high low depression (*y*-axis). Note. The word clouds on the left are represent young people and those in the right old people (*r* = 0.28). The upper word clouds represent high PHQ-9 scores and the lower word clouds low PHQ-9 scores (*r* = 0.76). See also footnote to Fig. 1.

**Fig. 5:**
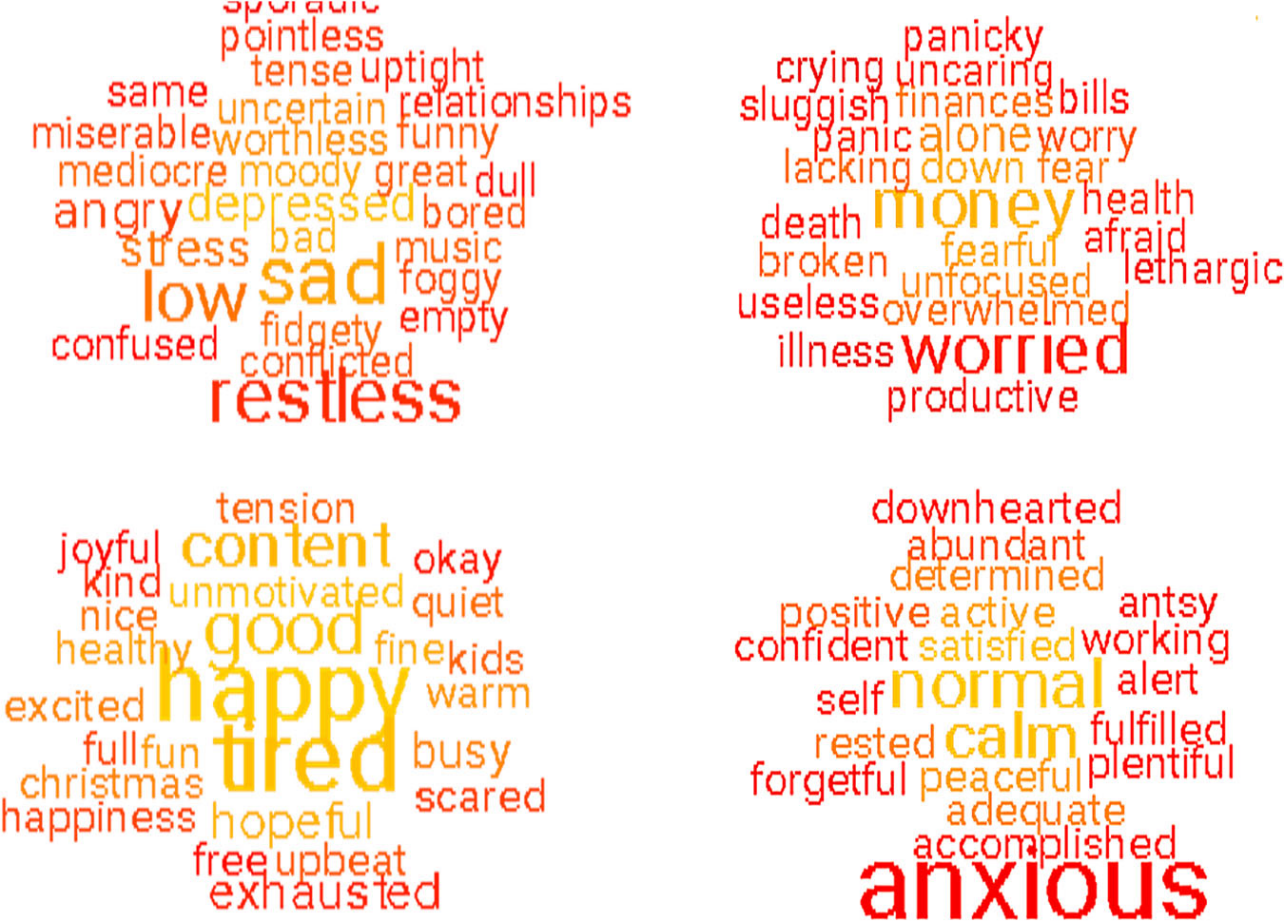
Word clouds indicative of young and old participants (*x*-axis) and high low anxiety (*y*-axis). Note. Same as Fig. 4, however, the upper word clouds represent high GAD-7 and the lower word clouds low GAD-7 (*r* = 0.71).

Word clouds show words indicative of young and old people (on the x-axis) with low or high for depression (on the *y*-axis for Fig. 4) and low or high anxiety (on the y-axis for Fig. 5). Rating scales and semantic measures of mental health were correlated with age (Table 3). Rating scales of depression (PHQ-9) and anxiety (GAD-7) correlated negatively with age following the Bonferroni correction of multiple comparisons. Similar results were found for the corresponding semantic measures, based on training of these rating scales. Finally, we correlated the semantic measures, using the rating scales as covariates. The results show that the semantic measures of depression and anxiety still correlated with age following the corresponding rating scales as covariates.

**Table 3:** Age correlated with mental health measure.

Measure	Rating scale		Semantic measures		Semantic measures with rating scales covariates	
	*r*	*p*	*r*	*p*	*r*	*p*
Depression	−0.20	0.0000^a^	−0.13	0.0002^a^	−0.0800	0.0121^b^
Anxiety	−0.19	0.0000^a^	−0.10	0.0039^a^	−0.0700	0.0493^b^

Note. The four measures of mental health are represented on each row. The columns show Pearson correlation to age, and the *p* values indicate the probability that the correlation is above zero.

^a^Significant following the Bonferroni correction for multiple comparison (*N* = 4).

^b^Significant without correction for multiple comparisons. Alpha was set to 0.05.

## Discussion

The aim of this article has been to investigate age differences in mental health using semantic representations generated from descriptive keyword responses to mental health questions. Indeed, the results demonstrated age differences; (1) middle-aged adults describe their mental health differently compared to younger adults; (2) for example, middle-aged adults emphasise depression and loneliness, whereas young adults list anxiety and money; (3) different semantic models are warranted for younger and middle-aged adults; (4) middle-aged participants described their mental health more accurately compared to young participants; (5) middle-aged adults have better mental health than younger adults as measured using semantic measures.

The first and second hypotheses addressed age differences to be found in the semantic representation. The age differences found in specifically semantic open-ended mental health questions is a novel discovery. Our data provides the possibility to summarise age-related themes linked to young and old people, using indicative words (see word cloud in Figs. 1, 2, 4, and 5). The young population lists words linking to aspects of social relationships, suggesting these are important for their mental health, while the older adults use words related to health, disease, death, insomnia, sadness and appetite.

Previous reports using more traditional rating scales have found that geriatric depression may emerge from neuronal age-related changes, and sometimes even neurodegenerative disease and cardiovascular changes in the brain^28^. This has given rise to selective rating scales for the elderly, such as the Geriatric Depression Scale^65^. Age differences in reported symptoms may, in part, be the result of generational differences regarding environmental factors such as personal circumstances (e.g., refs. ^19,20,33,66–68^). This explanation could be of particular importance as genetic factors potentially play a greater role in the emergence of depression and anxiety among younger adults^27,28^. The semantic open-ended question tool used in the current report may aid, speed up and facilitate proper diagnostic process regardless of a patient’s age in primary care context where expertise in geriatrics is less common.

The third hypothesis assumes that younger and older people may require different semantic prediction models. The present findings suggest that different prediction models are needed for younger and older adults. However, the model most appropriate for middle-aged adults was also better fitted to the data from the younger participants. We propose that the semantic data contains sufficient information for generating reasonable predictions in data from both younger and middle-aged adults. Middle-aged adults often out-perform younger adults in language skills^69^. The elderly has more advanced semantic networks as life experience may, in part, mediate such effects. Future studies focussing on an elderly sample may benefit from the assessment of language skills as a potential moderator of the effects reported herein.

The fourth hypothesis examined how well the semantic representation could predict rating scales depended both on whether the prediction models were based on younger or older adults. The prediction model of several ratings scales yielded higher accuracy when training was based on the older participants. A possible interpretation of this is that middle-aged adults are better at expressing their mental health in free words than younger adults. This finding was true, both when the data was evaluated on the younger and the middle-aged groups. This suggests that the finding cannot be easily explained with the notion that younger adults are less careful when responding to surveys. Sloppy answers would have generated less accurate rating scales, leading to the poorest predictability when applying the young model to the young dataset. In contrast, we found an interaction effect between the age group that the model was trained on and the age group that it was evaluated on, possibly suggesting a difference in semantic models for young and old. Overall, this suggests an interpretation that the older adults generated more informative descriptive keywords of their mental health than their younger counterparts.

Hypothesis 5 states that mental health varies in younger and older adults. According to the present study, older age was associated with lower levels of depression, which aligns with previously reported findings (18–29 years) in Villarroel et al.^70^, who discovered this was the case for both the rating scales and the semantic measures. Interestingly, these findings remain significant for the semantic measures, even after controlling for the effect of more traditional rating scales such as PHQ-9. This may indicate that the semantic measure of depression and anxiety provides additional information to the results of the rating scales.

Language is the natural way for people to communicate their mental state. Nevertheless, the dominating method of measuring psychological constructs are rating scales. A possible reason for this is that language has been difficult to quantify. Recent developments in natural language processing provide unprecedented opportunities for measuring language, with the possible application to mental health and ageing. There are several advantages with QCLA:

*Language is the natural* way for people to communicate their mental state. Sikström et al.^71^ showed that people prefer to describe their mental health using written language responses, as they found this method to be more precise and they are able to elaborate on their responses. Additionally, it was the preferred way to communicate with mental health professionals compared to rating scales. However, when rating scales were preferred, this was due to their ease and speed of use.

*Language base measure of mental health has high validity*. When mental health is measured using computational methods using words generated to describe mental health, there is evidence of a high correlation with validated scales of depression and anxiety^9^. Furthermore, combining free text and word responses about harmony and satisfaction using transformer-based models demonstrate, to our knowledge, the highest correlation yet between language responses and rating scales, which rivals the theoretical limits based on test–retest data (*r* = 0.84, *r*^2^ = 66).

*Language can be used to describe mental health constructs*. Rating scales are defined by researchers and provide a fixed measure of scale. In contrast, the QCLA approach allows for a data-driven measure of constructs, where data from a specific group of participants (i.e., culture, age, etc.) can be used to describe constructs. This definition can subsequently be visualised in a word cloud. We believe that this provides a more dynamic and natural way of thinking about mental constructs, as the scales of constructs are generated from data in a particular context.

*Computational analysis of language can be used for clinical assessments*. In combination with machine learning, the semantic mental health constructs can estimate age-specific mental health trajectories. Such algorithms may contribute to more efficient healthcare treatments, or may even serve as a means for notifying healthcare personnel or family members about how to act on subclinical symptoms and how to best support individuals with mental health problems.

*Personal assessment*. One major strength of the open-ended measure of mental health is that the participants describe their mental health status in their own words. This measure promotes ecological validity to a greater extent, as the responses are closer to their personal communication style and real-life context when compared to traditional rating tools, such as Likert scales, based on fixed items. Furthermore, open-ended questions can counteract the effect of reporting bias when assessing mental health. Self-reported information from traditional questionnaires may contain social desirability biases^72^, which can escalate or underestimate the studied effects of mental health.

The present results should be interpreted in the context of some limitations of QCLA. First, the study suffers from limited generalisability due to the non-random recruitment procedure. Second, another limitation is the associative nature of the current study, which precludes making direct inference about causality due to the lack of experimental control. Third, the sample consisted of a small proportion of old adults. There is a demand for future studies to focus on this age group in order to conclude differences of language usage and AI models to describe mental health in the elderly. Therefore, our results would benefit from future replications to increase the generalisability.

In conclusion, combining latent semantic estimates with machine learning methods may provide new opportunities to discriminate, model, and describe mental health in older and younger adults. Together, these methodologies may provide greater accuracy and precision in the evaluation of mental health across the adult lifespan.

## Supplementary information

Supplementary Materials

## Data Availability

The data is not publicly available as it includes sensitive text data; however, requests for the data can be submitted to the corresponding author.

## Code availability

The data analysis was conducted using the online software for statistical analysis of text, SemanticExcel that can be accessed on semanticexcel.com^73^. For code, see https://github.com/sverkersikstrom/semanticCode.
