# Supplementary material for: Assessment of depression and anxiety in young and old with a question-based computational language approach
Source: Npj Ment Health Res. 2023 Jul 24;2:11. doi: 10.1038/s44184-023-00032-z (PMC10955843; doi:10.1038/s44184-023-00032-z)
Supplement: Supplementary file 1 — Supplementary Materials [file 44184_2023_32_MOESM1_ESM.pdf]

Supplementary Information: Validation of the SDAS for MDD and GAD

The *SDAS symptoms focus* is based on Yes and No questions about symptoms described in the DSM-5 for MDD and GAD (see questions and question codes below). The answers were computed as described in the DSM-5:

MDD: Respondents answering Yes to either SDAS_MDD_1 or SDAS_MDD_2 – and together answering Yes to at least 5 of SDAS_MDD_1 – SDAS_MDD_9 are categorized as depressed.

GAD: Respondents answering Yes to SDAS_GAD_1 – SDAS_GAD_4, and three or more of SDAS_GAD_5 – SDAS_GAD_10.

Our study comprised 88 participants (Female = 50; Male = 35, Gender variant/Non-conforming = 1, Transgender Male = 1, Other = 1), with a mean age of 36.6 (SD = 12.6, range = 18 – 65), who completed the study. In random order they answered the SDAS and participated in a video call MINI interview for MDD and GAD, conducted by an experienced clinical psychologist. Table Supplementary Table 1 presents the agreement between the SDAS and the MINI interview.

Supplementary Table 1. Agreement between SDAS and MINI

	**SDAS – MINI interview**	
**Measure**	**MDD**	**GAD**
**kappa**	.7500	.5987
**sensitivity**	.9024	.7500
**specificity**	.8511	.8462
**balanced accuracy**	.8768	.7981
